# Sex-related differences in platelet aggregation: rebuttal to reply

**DOI:** 10.1093/ehjcvp/pvaf059

**Published:** 2025-08-04

**Authors:** Marco Cattaneo

**Affiliations:** Dipartimento di Scienze della Salute, Università degli Studi di Milano, Milano 20142, Italy; Fondazione Arianna Anticoagulazione, Via Paolo Fabbri, 1/3, Bologna 40138, Italy

**Keywords:** Sex, Platelet aggregation, Aspirin, Clopidogrel, Hematocrit, Laboratory artefacts

The reply by Galli *et al*.^1^ to my letter^2^ commenting their study^3^ of sex differences in platelet aggregation failed to address my comments properly, probably because they were expressed with insufficient clarity. I wish to clarify better my insight into the issue of sex difference in platelet aggregation addressed in the study by Galli *et al*.^3^

In their reply, the Authors mostly focused on the results of the study by Kelton *et al*.^4^ (quoted in my letter^2^), which they erroneously perceived to be different from those of their study, which showed that platelet aggregation, when studied by light-transmission aggregometry in platelet-rich plasma (PRP) anticoagulated with fixed volumes of citrate, is higher in women than men.^3^ One of the main criticisms raised by the Authors was that the results of the small study by Kelton *et al*. (22 subjects) cannot be compared with their large study (1483 subjects), whose sample size allowed the detection of statistically significant differences between sexes. The perceived presence of discrepancies between the Authors’ and Kelton’s studies epitomizes the most relevant misunderstanding that influenced the essence of the Authors’ reply.^1^ Indeed, the results of the study performed by Kelton *et al*. in their highly specialized laboratory, far from contrasting those reported by Galli *et al*.,^3^ perfectly matched them: the extent of platelet aggregation induced by adenosine diphosphate at concentrations ranging from 0.5 to 5 μM (comprising the 2 μM concentration used by Galli *et al*.^3^) was significantly higher in women than men.^4^ To the best of my knowledge, all the similar studies published in the last 50 years, including the large Northwick Park Heart Study (958 subjects) and Framingham Study (3429 subjects), reported similar results.^2^ Therefore, the concept that *in vitro* platelet aggregation is higher in women than men is well established and accepted by the scientific community, although the real reason for this discrepancy is apparently not as widely known.

The great importance of the study by Kelton *et al*. lies actually in its elegant demonstration that the observed sex differences in platelet aggregation are determined by an artefact caused by the increase in volume distribution of citrate in women’s blood samples, as a consequence of their lower haematocrit levels compared with men’s blood samples. The study by Kelton *et al*., published in 1980, may be considered old (which is not in itself a flaw), but certainly not dated, as the Authors alluded in their reply,^1^ because it was methodologically very sound and its results were confirmed by subsequent studies, some of which were quoted in my letter.^2^ Therefore, the focus of my comment has never been on the validity of the results of the study by Galli *et al*.,^3^ which are perfectly in line with all previously published studies and also with my personal experience,^2^ but, rather, on the real biological relevance of the observed sex difference in platelet aggregation, which is to be ascribed to an *in vitro* artefact.^2^

The relevance of the haematocrit- and citrate-dependent artefact affecting the results of *in vitro* platelet aggregation applies not only to differences in sex, but also to any conditions associated with abnormal haematocrit levels. Among these, ageing could be considered, as the lower haematocrit of elderly subjects could hypothetically partially contribute to the known ‘hyper-aggregability’ of their platelets^5^ and possibly explain the observation by Galli *et al*.^1,3^ that womens’ ‘hyper-aggregability’ is particularly pronounced in subjects >50 years of age. The haematocrit-adjusted citrate concentration used in parallel with standard fixed citrate concentration in the same subjects allowed Kelton *et al*.^4^ to unravel the artefact in their small sample-size study. Although haematocrit-adjusted citrate concentration is not recommended for routine use,^6^ evaluation of results of platelet aggregation in citrate PRP must not overlook the potential interference of abnormal haematocrit levels. This is particularly relevant in the practice of antiplatelet treatment monitoring, where small differences in borderline results could assign the same patient to the group of ‘high’ or ‘low’ on treatment platelet reactivity, which would condition the implementation of different treatment strategies.^7^ For example, in 475 clopidogrel-treated patients who were monitored by VerifyNow (which uses citrate-anticoagulated blood), the prevalence of high on-treatment platelet reactivity decreased from 54.5% to 41.9% after correction of the results on the basis of the haematocrit levels.^8^ This and other methodological flaws might be responsible for the failure of platelet function tests (PFT) monitoring of antiplatelet therapy,^7^ which was demonstrated by a meta-analysis restricted to randomized controlled trials with correct and homogeneous study designs, which guided antiplatelet therapy uniquely based on the results of PFT.^9^

## Data Availability

No data were generated or analysed for this article.

## References

[1] Galli M, Angiolillo DJ, Pulcinelli FM. Reply: sex-related variations in platelet reactivity. Eur Heart J Cardiovasc Pharmacother 2025;11:520–521. 40685252 10.1093/ehjcvp/pvaf054 PMC12450596

[2] Cattaneo M. Sex-related differences in platelet aggregation. Eur Heart J Cardiovasc Pharmacother 2025;11:518–519. 40671586 10.1093/ehjcvp/pvaf052 PMC12450590

[3] Galli M, Terracina S, Schiera E, De Corci S, Sangiorgi D, Mancone M, Frati L, Sciarretta S, Angiolillo DJ, Pulcinelli FM. Sex-related variations in platelet reactivity in presence or absence of antiplatelet therapy. Eur Heart J Cardiovasc Pharmacother 2025;11:509–517. 40366913 10.1093/ehjcvp/pvaf034 PMC12450592

[4] Kelton JG, Powers P, Julian J, Boland V, Carter CJ, Gent M, Hirsh J. Sex-related differences in platelet aggregation: influence of the hematocrit. Blood 1980;56:38–41. 7388180

[5] Le Blanc J, Lordkipanidzé M. Platelet function in aging. Front Cardiovasc Med 2019;6:Article 109. 31448291 10.3389/fcvm.2019.00109 PMC6692461

[6] Cattaneo M, Cerletti C, Harrison P, Hayward CP, Kenny D, Nugent D, Nurden P, Rao AK, Schmaier AH, Watson SP, Lussana F, Pugliano MT, Michelson AD. Recommendations for the standardization of light transmission aggregometry: a consensus of the working party from the platelet physiology subcommittee of SSC/ISTH. J Thromb Haemost 2013;11:1183–1189. 10.1111/jth.12231 23574625

[7] Cattaneo M, Squizzato A, Birocchi S, Podda GM. Guided anti-P2Y12 therapy in patients undergoing percutaneous coronary intervention. Thromb Haemost 2024;124:595–597. 37992750 10.1055/a-2216-5263

[8] Tanaka K, Matsumoto S, Ainiding G, Nakahara I, Nishi H, Hashimoto T, Ohta T, Sadamasa N, Ishibashi R, Gomi M, Saka M, Miyata H, Watanabe S, Okata T, Sonoda K, Koge J, Iinuma KM, Furuta K, Nagata I, Matsuo K, Matsushita T, Isobe N, Yamasaki R, Kira JI. PON1 Q192R is associated with high platelet reactivity with clopidogrel in patients undergoing elective neurointervention: a prospective single-center cohort study. PLoS One 2021;16:e0254067. 34351918 10.1371/journal.pone.0254067 PMC8341610

[9] Birocchi S, Rocchetti M, Minardi A, Podda GM, Squizzato A, Cattaneo M. Guided anti-P2Y12 therapy in patients undergoing PCI: three systematic reviews with meta-analyses of randomized controlled trials with homogeneous design. Thromb Haemost 2024;124:482–496. 37549688 10.1055/a-2149-4344

